# Effect of Sodium Hypochlorite Exposure on Cyclic Fatigue Resistance of Two Heat-Treated Nickel-Titanium Rotary Instruments: An In Vitro Study

**DOI:** 10.7759/cureus.117590

**Published:** 2026-10-07

**Authors:** Parvaze Ahmad Parvaze, Khalid Amin Lone, Shafiat Ullah Khateeb, Aamir Rashid Pura, Ajaz Masoodi

**Affiliations:** 1 Department of Restorative Dentistry and Endodontics, Primary Health Care Corporation, Doha, QAT; 2 Department of Endodontics, Central Security Hospital, Riyadh, SAU; 3 Department of Restorative Dental Sciences, College of Dentistry, King Khalid University, Abha, SAU; 4 Department of Conservative Dentistry and Endodontics, Government Dental College and Hospital, Srinagar, IND

**Keywords:** equipment failure, fatigue, nickel-titanium, root canal preparation, sodium hypochlorite

## Abstract

Introduction

Heat-treated nickel-titanium (NiTi) rotary instruments are widely used for root canal preparation; however, their resistance to cyclic fatigue may be affected by alloy characteristics and exposure to endodontic irrigants. This study aimed to compare the cyclic fatigue resistance of ProTaper Gold (PTG) and EdgeTaper Platinum (ETP) instruments following single and repeated exposures to 5.25% sodium hypochlorite (NaOCl).

Materials and methods

This in vitro experimental study included 48 new rotary instruments, comprising 24 PTG instruments (50.0%) and 24 ETP instruments (50.0%). Each system was divided equally into distilled water control, single NaOCl exposure, and repeated NaOCl exposure groups, with eight (16.7%) instruments in each subgroup. The instruments were tested in a standardized 60degree curved artificial canal at 37°C. The primary outcome was the number of cycles to failure (NCF). Data were analyzed using two-way analysis of variance, Tukey's honestly significant difference test, and independent-sample t-tests.

Results

ETP demonstrated significantly greater cyclic fatigue resistance than PTG (main effect, p < 0.001; partial η² = 0.769). Exposure condition also significantly affected cyclic fatigue resistance (p < 0.001; partial η² = 0.431), whereas the instrument system × exposure condition interaction was not significant (p = 0.696; partial η² = 0.017). Compared with the control condition, single NaOCl exposure significantly reduced NCF (p = 0.003; Cohen’s d = 1.21), as did repeated exposure (p < 0.001; Cohen’s d = 2.04). The difference between single and repeated NaOCl exposures was also statistically significant (p = 0.002; Cohen’s d = 0.95). ETP demonstrated significantly greater cyclic fatigue resistance than PTG under all three exposure conditions (all p < 0.001).

Conclusion

ETP showed greater cyclic fatigue resistance than PTG, whereas 5.25% NaOCl exposure adversely affected the fatigue resistance of both systems.

## Introduction

Nickel-titanium (NiTi) rotary instruments have become an integral component of contemporary root canal preparation owing to their flexibility, superelasticity, and ability to maintain canal anatomy during mechanical instrumentation [1]. However, instrument separation remains an important procedural complication, particularly when files are subjected to repeated tensile and compressive stresses within curved root canals [2]. Cyclic fatigue is one of the principal mechanisms contributing to instrument fracture, and it occurs when a rotating instrument repeatedly passes through a region of maximum curvature. Cyclic fatigue resistance of NiTi instruments is influenced by several factors, including the alloy composition, manufacturing process, instrument geometry, taper, rotational speed, and canal curvature [3,4]. 

Advances in thermomechanical processing have resulted in heat-treated NiTi alloys with altered phase-transformation characteristics and improved mechanical performance [5]. Heat-treated systems such as ProTaper Gold (PTG) and EdgeTaper Platinum (ETP) have been developed to provide greater flexibility and resistance to mechanical fatigue compared to conventional NiTi instruments [6]. Nevertheless, the clinical environment exposes endodontic instruments to irrigating solutions that may interact with their metallic surfaces and potentially alter their mechanical properties [7].

Sodium hypochlorite (NaOCl) is the most widely used irrigating solution in endodontic treatment because of its antimicrobial activity and ability to dissolve organic tissues [8]. However, its chemical and potentially corrosive effects may influence the surface characteristics and mechanical behavior of NiTi instruments [9]. Experimental studies have demonstrated that exposure to NaOCl can reduce cyclic fatigue resistance in some NiTi systems, with the magnitude of the effect influenced by the concentration, temperature, and duration of NaOCl exposure, and metallurgical characteristics of the instrument [9,10]. Furthermore, a systematic review and meta-analysis of in vitro studies reported an overall tendency toward reduced cyclic fatigue resistance following exposure to NaOCl, although the effect varied considerably among different instrument systems [11]. Importantly, relatively limited evidence is available on the influence of repeated exposure to clinically relevant concentrations of NaOCl on contemporary heat-treated instruments.

Therefore, the present in vitro study was designed to evaluate whether NaOCl exposure and the instrument system independently influence cyclic fatigue resistance and whether an interaction exists between these factors. The objectives were to compare the number of cycles to failure (NCF) between the two instrument systems under each exposure condition and determine the effect of single versus repeated NaOCl exposure on cyclic fatigue resistance. We hypothesized that cyclic fatigue resistance would differ between PTG and ETP instruments, that exposure to 5.25% NaOCl would reduce cyclic fatigue resistance, and that the effect of NaOCl exposure would differ according to the instrument system.

## Materials and methods

Study design and ethical considerations

This in vitro laboratory experimental study was conducted in the Department of Conservative Dentistry and Endodontics, Government Dental College and Hospital, Srinagar, India, over a period of five months between June 2022 and November 2022. This study involved commercially manufactured NiTi rotary endodontic instruments and laboratory-based experimental procedures. No human participants, biological specimens, animals, patient records, or identifiable clinical data were included, and therefore, ethical approval was not required for this study. All experimental procedures were performed under standardized laboratory conditions, and the study protocol was followed consistently throughout the experimental period.

Sample selection

A total of 48 new, unused, heat-treated NiTi rotary instruments were included. Twenty-four PTG (Dentsply Sirona, York, Pennsylvania, USA) and 24 ETP (EdgeEndo, Albuquerque, New Mexico, USA) instruments were evaluated. Instruments corresponding to size #25 with a 0.06 taper were selected for both systems. PTG incorporates gold-wire heat-treatment technology, whereas ETP is manufactured using FireWire heat-treated NiTi alloy [6]. Before inclusion, all the instruments were examined under a stereomicroscope at 20× magnification. Instruments showing visible manufacturing defects, surface irregularities, deformations, or other structural abnormalities were excluded.

Sample size calculation

An a priori sample-size calculation was performed using the G*Power software, version 3.1 (Heinrich Heine University, Düsseldorf, Germany). Because the primary analysis was a two-way factorial analysis of variance (ANOVA), the sample size was determined using an F-test for fixed-effects ANOVA, corresponding to a 2 × 3 factorial design with two instrument systems and three exposure conditions (six experimental cells). The calculation was based on an assumed ANOVA effect size of f = 0.70, an alpha level of 0.05, and statistical power of 80% (1 − β = 0.80). The resulting minimum sample requirement was 48 instruments, corresponding to eight instruments per experimental cell. Accordingly, 24 instruments were allocated to each instrument system, with eight instruments assigned to each of the three exposure conditions. This allocation provided equal and balanced representation across all six experimental cells.

Group allocation

The 48 instruments were first stratified according to the instrument system, with 24 PTG and 24 ETP instruments. Within each instrument system, the 24 instruments were randomly allocated to three exposure conditions: no-exposure control, single 5.25% NaOCl exposure, and repeated 5.25% NaOCl exposure, with eight instruments assigned to each condition. A computer-generated random-number sequence was used for the allocation, and the allocation sequence was prepared before commencement of the exposure procedures. Because this was an in vitro laboratory study involving individually identifiable instruments rather than human participants, formal allocation concealment was not considered applicable. The final allocation was balanced, with each exposure condition containing eight PTG and eight ETP instruments. The control subgroup received no chemical exposure and was tested immediately without immersion. The single-exposure subgroup consisted of instruments immersed in 5.25% NaOCl maintained at 37°C for 5 min. Repeated-exposure subgroup consisted of instruments subjected to three consecutive 5-min immersions in 5.25% NaOCl maintained at 37°C, with freshly prepared solution used for each exposure cycle. Thus, each exposure condition contained 16 instruments overall, comprising eight PTG and eight ETP instruments, resulting in equal and balanced allocation across all instrument systems and exposure conditions (Figure 1).

**Figure 1:**
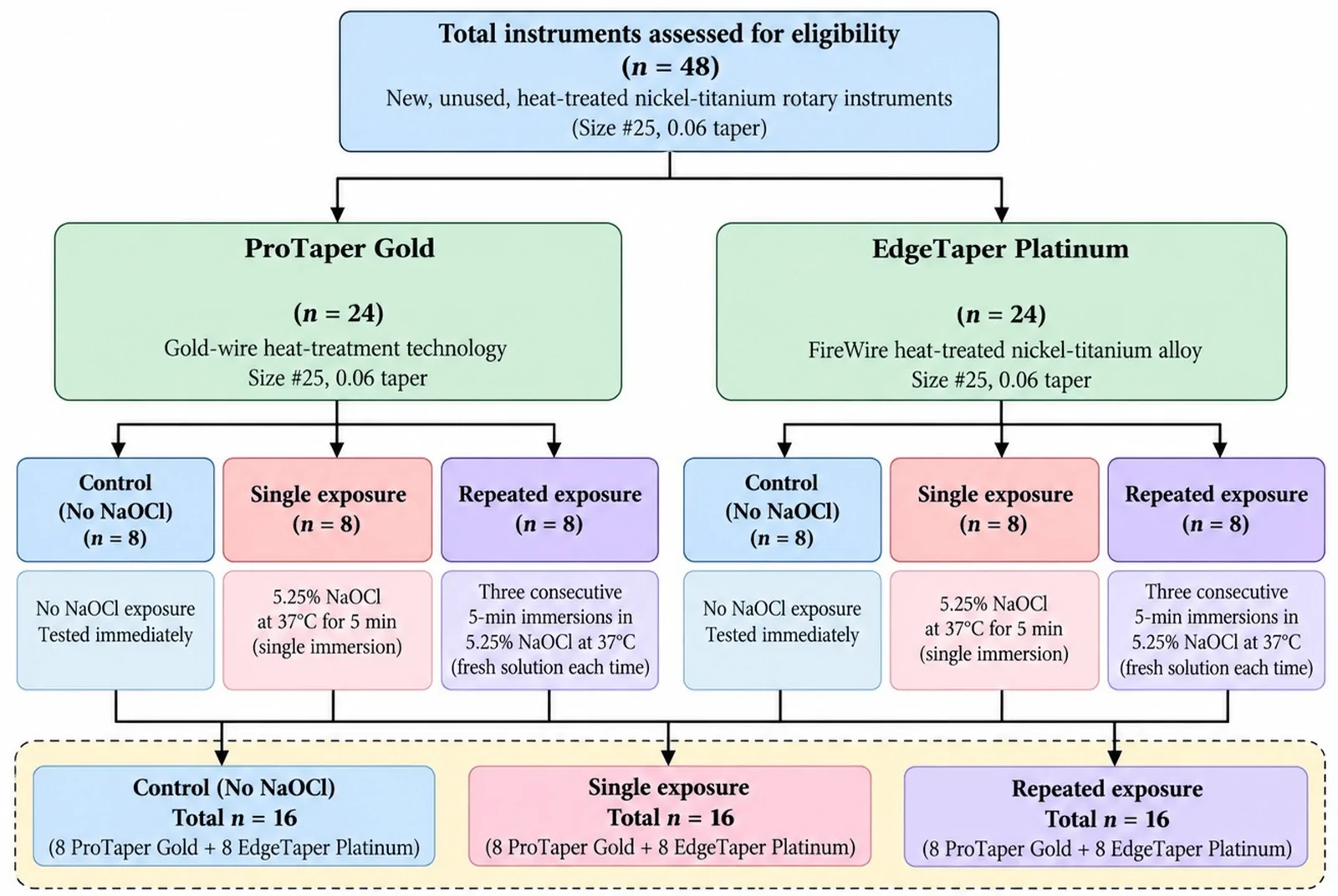
Study flowchart and instrument group allocation. The image was created using Canva (Canva Pvt. Ltd., Australia).

NaOCl exposure protocol

A commercially available 5.25% NaOCl solution (Hypochlor Forte; SafeEndo Dental India Pvt. Ltd., Vadodara, Gujarat, India) was used in the experimental exposure groups. The solution was maintained at 37 ± 1°C in a thermostatically controlled water bath. A concentration of 5.25% was selected as the clinically relevant concentration specified in the study protocol. For the single-exposure group, the working portion of each instrument, extending from the tip to the 16-mm mark and excluding the shaft, was completely immersed in the NaOCl solution for 5 min. For the repeated-exposure group, the procedure was performed three consecutive times, with each cycle lasting 5 min, using freshly prepared NaOCl solution. After each exposure, the instruments were rinsed with distilled water for 30 s to remove residual NaOCl and gently air-dried before cyclic fatigue testing. The control instruments were not exposed to NaOCl and were tested without immersion.

The 5-min exposure duration was selected based on a previously published laboratory protocol evaluating the effects of endodontic irrigating solutions on the cyclic fatigue resistance of NiTi instruments [12]. The repeated-exposure protocol consisted of three consecutive 5-min exposures using freshly prepared 5.25% NaOCl for each cycle. This design was intended to provide a standardized model for examining the effect of repeated chemical exposure and to permit comparison between single and cumulative exposure conditions under controlled laboratory conditions. The protocol was not intended to reproduce a specific clinical instrument-reuse schedule.

Artificial canal and cyclic fatigue testing apparatus

Cyclic fatigue testing was performed using a custom-fabricated stainless steel artificial canal block. The artificial canal had a 60degree angle of curvature and a 5-mm radius of curvature, with the center of curvature positioned 5 mm from the instrument tip. The canal had a diameter of 1.5 mm and was machined to accommodate size #25 instruments. The artificial canal block was positioned inside a thermostatically controlled water bath containing distilled water, maintained at 37 ± 1°C throughout the test. A small amount of a standardized endodontic lubricant, Glyde File Prep (Dentsply Sirona; Charlotte, North Carolina, USA), was applied to the canal to reduce friction during instrument rotation.

Cyclic fatigue testing procedure

Each instrument was securely mounted on a torque-controlled endodontic motor using a contra-angle handpiece. An X-Smart Plus endodontic motor (Dentsply Sirona; Charlotte, North Carolina, USA) was used. The instruments were operated at 300 rpm using the manufacturer's recommended torque settings. No standardized axial pecking motion was applied during testing; therefore, no axial movement amplitude or pecking frequency was specified. Each instrument was introduced into the standardized artificial canal and rotated continuously until fracture. The dynamic testing configuration permitted the instrument to move freely within the canal during rotation. The time to fracture was recorded in seconds using a digital stopwatch. The NCF was calculated as follows:

\[ \mathrm{NCF} = \frac{\text{Time to fracture (s)} \times \text{Rotational speed (rpm)}}{60} \]

where NCF is the number of cycles to failure. After the fracture, the length of the separated fragment was measured using a digital caliper to verify that the fracture had occurred within the curved portion of the artificial canal. Instruments that fractured outside the predefined curved segment were excluded and replaced according to the experimental protocol. The single-exposure condition was included as a standardized laboratory exposure condition to determine whether a defined NaOCl challenge was associated with measurable changes in cyclic fatigue resistance. The repeated-exposure condition was included to examine whether cumulative exposure produced an additional effect compared with a single exposure. These exposure conditions were intended for controlled experimental comparison and were not designed to reproduce the complexity of clinical reuse, including biological factors such as saliva, biofilm, dentinal contact, and other intra-canal conditions.

Standardization and quality control

To minimize experimental variability, all instruments were new, unused, and underwent standardized inspections before testing. The instrument size, taper, NaOCl concentration, exposure duration, exposure temperature, canal geometry, testing temperature, rotational speed, and torque settings were standardized throughout the experiment. The same artificial canal configuration, endodontic motor, testing conditions, and measurement procedures were used for all the specimens. Fresh NaOCl solution was used for each repeated exposure cycle, and all the exposed instruments underwent the same rinsing and drying procedure.

Outcome measure

The primary outcome was the NCF, calculated from the recorded time to fracture and rotational speed of the instrument. Higher NCF values indicate greater cyclic fatigue resistance.

Statistical analysis

All statistical analyses were performed using IBM SPSS Statistics for Windows (version 25.0; IBM Corp., Armonk, New York, USA). As no instrument was fractured outside the curved canal, all 48 instruments were included in the analysis. The normality of the NCF data was assessed using the Shapiro-Wilk test, and homogeneity of variances was evaluated using Levene's test. Both tests supported the assumptions for the parametric analysis (p > 0.05). Continuous data are expressed as mean ± standard deviation. A two-way ANOVA was performed to assess the main effects of the instrument system (PTG versus ETP), exposure conditions (distilled water control, single 5.25% NaOCl exposure, and repeated 5.25% NaOCl exposure), and the interaction between the instrument system and exposure condition. For significant main effects, Tukey's honestly significant difference (HSD) test was used for post-hoc pairwise comparisons. Also, independent sample t-tests were used to compare the two instrument systems within each exposure condition. Effect sizes were reported using Cohen's d for pairwise comparisons and partial eta squared (η²p) for ANOVA variance. Statistical significance was set at p < 0.05.

## Results

A total of 48 rotary instruments were evaluated, comprising 24 (50.0%) PTG instruments and 24 (50.0%) ETP instruments. Within each instrument system, eight (33.3%) instruments were allocated to each of three exposure conditions: distilled water control, single exposure to 5.25% NaOCl, and repeated exposure to 5.25% NaOCl. Overall, each exposure condition included 16 (33.3%) instruments, demonstrating equal distribution across the study groups (Table 1).

**Table 1:** Distribution of instruments according to instrument system and exposure condition. Data are presented as frequencies and percentages. NaOCl: sodium hypochlorite

Instrument	Distilled water	5.25% NaOCl single exposure	5.25% NaOCl repeated exposure	Total
ProTaper Gold	8 (50%)	8 (50%)	8 (50%)	24 (50%)
EdgeTaper Platinum	8 (50%)	8 (50%)	8 (50%)	24 (50%)
Total	16 (100%)	16 (100%)	16 (100%)	48 (100%)

Comparison of cyclic fatigue resistance

The mean NCF was consistently higher for ETP than for PTG under all three exposure conditions. A two-way ANOVA demonstrated a statistically significant main effect of the instrument system on cyclic fatigue resistance (F = 139.47, p < 0.001; partial η² = 0.769). A significant main effect of the exposure condition was also observed (F = 15.93, p < 0.001; partial η² = 0.431). However, the interaction between the instrument system and exposure condition was not statistically significant (F = 0.364, p = 0.696; partial η² = 0.017), indicating that the influence of NaOCl exposure on cyclic fatigue resistance did not differ significantly between the two instrument systems (Tables 2-3).

**Table 2:** Number of cycles to failure according to instrument system and exposure condition. Data are presented as mean ± standard deviation (SD). Higher NCF values indicate greater cyclic fatigue resistance. N: number of samples in the group; n: number of samples in subgroups; NCF: number of cycles to failure; NaOCl: sodium hypochlorite

Exposure condition	ProTaper Gold (PTG) N = 24	EdgeTaper Platinum (ETP) N = 24
	Mean NCF ± SD	Mean NCF ± SD
Distilled water (control), n = 8/group	939.4 ± 176.9	1664.5 ± 285.9
5.25% NaOCl, single exposure, n = 8/group	659.4 ± 111.8	1422.8 ± 246.6
5.25% NaOCl, repeated exposure, n = 8/group	571.1 ± 172.3	1211.8 ± 209.8

**Table 3:** Two-way analysis of variance for the effects of instrument system and exposure condition on cyclic fatigue resistance. A two-way ANOVA was used to assess the main effects of instrument system and exposure condition and their interaction. η²: eta squared represents the effect size for each ANOVA effect. *p < 0.05 is considered statistically significant. ANOVA: analysis of variance

Source of variation	Sum of squares	Degree of freedom	Mean square	F value	p-value	Partial η²
Instrument system	60,44,657	1	60,44,657	139.5	< 0.001*	0.769
Exposure condition	13,81,057	2	6,90,528	15.93	< 0.001*	0.431
Instrument system × exposure condition	31,527	2	15,764	0.364	0.696	0.017

Effect of NaOCl exposure

Tukey's HSD post-hoc analysis demonstrated significantly lower cyclic fatigue resistance following a single exposure to 5.25% NaOCl compared to the distilled water control (p < 0.001; Cohen's d = 1.21). The difference was more pronounced when the repeated-exposure condition was compared to the control (p < 0.001; Cohen's d = 2.04). The difference between single and repeated NaOCl exposures was also statistically significant (p = 0.002; Cohen's d = 0.95). These findings indicate that NaOCl exposure was associated with a significant reduction in cyclic fatigue resistance, with repeated exposure producing a further significant reduction compared with single exposure (Table 4).

**Table 4:** Pairwise comparison of cyclic fatigue resistance between exposure conditions. Pooled analysis across both instrument systems (ProTaper Gold and EdgeTaper Platinum). Data are presented as mean differences in the number of cycles to failure (NCF). Pairwise comparisons were performed using Tukey's honestly significant difference (HSD) post-hoc test following two-way ANOVA. Cohen's d represents the standardized effect size. *p < 0.05 is considered statistically significant. Data were pooled across both instrument systems (ProTaper Gold and EdgeTaper Platinum; n = 16 per exposure condition) because the two-way ANOVA revealed a non-significant interaction between instrument system and exposure condition. NaOCl: sodium hypochlorite; ANOVA: analysis of variance

Pairwise comparison	Mean difference (NCF)	t-value	p-value	Cohen's d
Distilled water vs. 5.25% NaOCl (single)	260.9	6.44	< 0.001*	1.21
Distilled water vs. 5.25% NaOCl (repeated)	460.6	10.87	< 0.001*	2.04
5.25% NaOCl (single) vs. 5.25% NaOCl (repeated)	199.7	3.19	0.002*	0.95

Comparison between instrument systems

ETP demonstrated a significantly greater cyclic fatigue resistance than PTG under each exposure condition. The between-system differences were statistically significant for the distilled water control, single NaOCl exposure, and repeated NaOCl exposure (all p < 0.001). The corresponding effect sizes were large, with Cohen's d values of 3.04, 3.78, and 3.27, respectively, indicating a consistently substantial difference in cyclic fatigue resistance favoring ETP (Table 5).

**Table 5:** Pairwise comparison of cyclic fatigue resistance between instrument systems according to exposure condition. Data are presented as mean differences in the number of cycles to failure (NCF), calculated as EdgeTaper Platinum (ETP) minus ProTaper Gold (PTG). Independent samples t-tests were used to compare the two instrument systems within each exposure condition. Cohen's d represents the standardized effect size, and *p < 0.05 is considered statistically significant. NaOCl: sodium hypochlorite

Exposure condition	Mean difference (NCF)	t-value	p-value	Cohen's d
Distilled water (ETP-PTG)	725.1	21.94	< 0.001*	3.04
5.25% NaOCl (single) (ETP-PTG)	763.4	23.67	< 0.001*	3.78
5.25% NaOCl (repeated) (ETP-PTG)	640.7	20.18	< 0.001*	3.27

## Discussion

The findings of the present study indicate that the mechanical behavior of heat-treated NiTi instruments is influenced by both the instrument system and the exposure to NaOCl. The consistently greater cyclic fatigue resistance observed with ETP than with PTG is in agreement with the findings of previous comparative investigations [13-15]. Jamleh et al. [13] reported superior cyclic fatigue resistance for ETP compared with PTG under simulated body temperature conditions, whereas Weyh and Ray [14] similarly demonstrated greater fatigue resistance for ETP in direct comparisons with PTG. More recent systematic review evidence has also demonstrated an overall advantage for ETP, although substantial heterogeneity among studies indicates that the magnitude of this difference is influenced by the experimental conditions [15].

Although comparative evidence regarding the cyclic fatigue resistance of ETP and PTG has recently been synthesized in a systematic review and meta-analysis [15], the available evidence primarily addresses differences between the two instrument systems under the experimental conditions reported in individual studies. Importantly, the effect of repeated exposure to NaOCl on these two contemporary heat-treated systems remains insufficiently characterized. The present study was therefore designed to address this specific gap by evaluating the independent effects of instrument system and 5.25% NaOCl exposure condition, including single and repeated exposure, on cyclic fatigue resistance under standardized laboratory conditions. In addition, the study evaluated the interaction between instrument system and exposure condition to determine whether the effect of NaOCl exposure differed between the two systems.

The superior fatigue behavior of ETP may be related to differences in thermomechanical processing, alloy characteristics, and instrument geometry. Although both systems undergo proprietary heat treatment, the FireWire technology used for ETP and the Gold-Wire treatment used for PTG produce different metallurgical characteristics and phase-transformation behaviors [16]. These differences can influence the flexibility, resistance to crack initiation, and ability of the instrument to withstand repeated tensile and compressive stresses during rotation. Importantly, cyclic fatigue resistance is not determined by heat treatment alone; cross-sectional design, taper, core dimensions, flute configuration, and the distribution of metal mass can also substantially affect mechanical performance [17]. Therefore, the observed superiority of ETP should be considered a combined consequence of its metallurgical and geometric characteristics rather than exclusively attributed to its heat-treatment technology.

Alcalde et al. [18] reported that edge-tapered platinum exhibited greater cyclic fatigue resistance than PTG when tested in a standardized curved artificial canal at body temperature. Their findings are particularly relevant because the experimental canal configuration was comparable to that used in the present study, thus strengthening the consistency of the observed differences between the two systems. Martins et al. [19] further demonstrated that heat-treated NiTi systems differ in their metallurgical characteristics, phase-transformation behavior, hardness, flexibility, and mechanical performance, indicating that differences in fatigue resistance cannot be attributed solely to heat treatment. These observations support the role of system-specific metallurgical and geometric characteristics in determining cyclic fatigue behavior.

Exposure to NaOCl was associated with reduced cyclic fatigue resistance in both systems. This finding is consistent with that in the broader literature, in which NaOCl has been shown to adversely affect the fatigue behavior of several NiTi instruments [10,11]. The proposed mechanisms include chemical interaction with the alloy surface, surface corrosion, formation or enlargement of microscopic defects, and the subsequent facilitation of crack initiation during cyclic loading [20]. These effects may become more relevant when instruments are exposed to relatively concentrated or heated irrigation solutions. A systematic review and meta-analysis found an overall tendency toward reduced cyclic fatigue resistance following NaOCl exposure, particularly under conditions involving higher concentrations and preheated solutions [11].

However, the effect of NaOCl was not uniform across all the NiTi instruments. Some investigations have reported minimal or non-significant changes following chemical exposure, particularly in certain heat-treated or controlled-memory alloys [11]. Such contrasting observations may reflect differences in the alloy composition, surface treatment, instrument geometry, solution concentration, temperature, exposure duration, and fatigue testing methodology. In particular, heat-treated instruments may respond differently to chemical exposure because their altered phase transformation characteristics can influence the mechanical behavior at body temperature. Consequently, the findings obtained from one instrument should not be automatically extrapolated to other NiTi systems.

The present findings are also consistent with those of Alfawaz et al. [12], who evaluated ETP and PTG under NaOCl and other irrigation conditions, using a standardized artificial canal at body temperature. Their findings supported the susceptibility of these instruments to changes in the chemical testing environment while also demonstrating differences between the two systems. Therefore, experimental evidence suggests that the influence of irrigation should be considered together with the metallurgical and geometric characteristics of the instrument, rather than as an isolated chemical effect.

From a clinical perspective, these findings are relevant when rotary instrumentation is performed in severely curved canals, in which cyclic fatigue is an important contributor to instrument separation. The selection of an instrument with a greater fatigue resistance may provide a mechanical advantage under demanding conditions. Nevertheless, laboratory cyclic fatigue performance cannot be equated directly with clinical fracture risk because clinical instrumentation involves simultaneous cyclic and torsional stresses, dentinal contact, anatomical irregularities, operator-related factors, and repeated clinical use. Appropriate glide-path preparation, avoidance of excessive apical pressure, controlled instrument use, and adherence to manufacturer recommendations remain important, regardless of the instrument system selected.

The principal limitations of this study include its in vitro design, use of a standardized artificial canal, evaluation of only two instrument systems, use of a single NaOCl concentration, and the controlled exposure protocol. Although the 5-min exposure duration and three consecutive exposure cycles provided a standardized model for comparing single and repeated NaOCl exposure, this protocol was not intended to reproduce a specific clinical instrument-reuse schedule. Clinical irrigant exposure may vary according to the duration and frequency of irrigant contact, irrigant concentration and temperature, instrument usage, and clinical operating conditions. Also, artificial canals cannot completely reproduce the anatomical and mechanical complexity of natural root canals. The controlled laboratory protocol also cannot reproduce the cumulative chemical and mechanical stresses encountered during clinical instrumentation. Therefore, the findings should be interpreted within the controlled experimental conditions of the present study rather than directly extrapolated to clinical instrument reuse or fracture risk. Further investigations that incorporate different irrigant concentrations and exposure durations, clinically representative repeated-use protocols, natural or extracted teeth, additional heat-treated instrument systems, and combined cyclic-fatigue and torsional testing would provide a more comprehensive understanding of instrument behavior under clinically relevant conditions.

## Conclusions

Under the standardized in vitro conditions of this study, ETP demonstrated greater cyclic fatigue resistance than PTG, while exposure to 5.25% NaOCl was associated with reduced cyclic fatigue resistance in both instrument systems. These findings indicate that instrument system and NaOCl exposure condition influence cyclic fatigue resistance under laboratory conditions. However, the results should not be directly extrapolated to clinical fracture risk, as clinical instrument separation is multifactorial and is influenced by anatomical, mechanical, chemical, and operator-related factors. Therefore, further studies under clinically representative conditions are warranted.
